# Next-Generation Sequencing Based HLA Typing: Deciphering Immunogenetic Aspects of Sarcoidosis

**DOI:** 10.3389/fgene.2018.00503

**Published:** 2018-10-25

**Authors:** Amit Kishore, Martin Petrek

**Affiliations:** Department of Pathological Physiology, Faculty of Medicine and Dentistry, Palacky University, Olomouc, Czechia

**Keywords:** HLA, next-generation sequencing, genotyping, immune diseases, disease association, sarcoidosis, molecular pathophysiology

## Abstract

Unraveling of the HLA-related immunogenetic basis of several immune disorders is complex due to the extensive HLA polymorphism and strong linkage-disequilibrium between HLA loci. A lack of in phase sequence information, a relative deficiency of high resolution genotyping including non-coding regions and ambiguous haplotype assignment make it difficult to compare findings across association studies and to attribute a causal role to specific HLA alleles/haplotypes in disease susceptibility and modification of disease phenotypes. Earlier, historical antibody and DNA-based methods of HLA typing, primarily of low resolution at antigen/alellic group levels, yielded “indicative” findings which were partially improved by high-resolution DNA-based typing. Only recently, next-generation sequencing (NGS) approaches based on deep-sequencing of the complete HLA genes combined with bioinformatics tools began to provide the access to complete information at an allelic level. Analyzing HLA with NGS approaches, therefore, promises to provide further insight in the etiopathogenesis of several immune disorders in which HLA associations have been implicated. These range from coeliac disease and rheumatological conditions to even more complex disorders, such as type-1 diabetes, systemic lupus erythematosus and sarcoidosis. A systemic disease of unknown etiology, sarcoidosis has previously been associated with numerous HLA variants and also other gene polymorphisms, often in linkage with the HLA region. To date, the biological significance of these associations has only partially been defined. Therefore, more precise assignments of HLA alleles/haplotypes using NGS approaches could help to elucidate the exact role of HLA variation in the multifaceted etiopathogenesis of sarcoidosis, including epigenetic mechanisms. NGS-based HLA analyses may be also relevant for defining variable clinical phenotypes and for predicting the disease course or the response to current/plausible novel therapies.

## Introduction

The human leucocyte antigen (HLA, also known as the major histocompatibility complex ‘MHC’) region, a 3.6 Mb segment of the human genome at 6p21.3, encodes more than 220 genes of diverse function, including 20 immune protein-coding genes. The codominant expression of HLA genes regulates the acquired immune response as HLA-class I and HLA-class II genes interact with T cell receptors (TCRs) of CD8^+^T_c_ and CD4^+^T_h_ cells, through cell-mediated and humoral immune response mechanisms, respectively. HLA gene expression is further activated by cytokines (e.g., IFN-γ, IL-4) produced during both innate and adaptive immune responses. The HLA region has been associated with numerous diseases, primarily, of an autoimmune or infectious nature (Shiina et al., 2009).

The HLA region is the most polymorphic in the human genome and contains six classical HLA genes: class I *HLA-A, -B*, and *-C* and class II *HLA-DRB1, -DQB1*, and *-DPB1* (Shiina et al., 2009; Trowsdale and Knight, 2013; Robinson et al., 2015). Polymorphisms in the extended HLA region also exhibit linkage disequilibrium (LD), including with specific classical HLA alleles. At the protein level, sequence variations in the peptide-binding cleft region of HLA-class I (α1 and α2 helixes) and HLA-class II (β1 domain) molecules (“HLA interacting domains”) interact with TCRs with differential antigen-binding properties, and thus may affect antigen presentation. The class I molecules are additionally sensed by receptor genes of NK cells (such as *killer-cell Ig-like receptors* ‘*KIR*’) and by cells of monocyte lineages (*leukocyte Ig-like receptor* ‘*LIR*’) (Shiina et al., 2009; Trowsdale and Knight, 2013). Thus, HLA and non-HLA genes, their intergenic variants and haplotypes within the HLA region exhibit polygenic loci and affect expression, peptide binding, and stability of the HLA-molecules.

**FIGURE 1:**
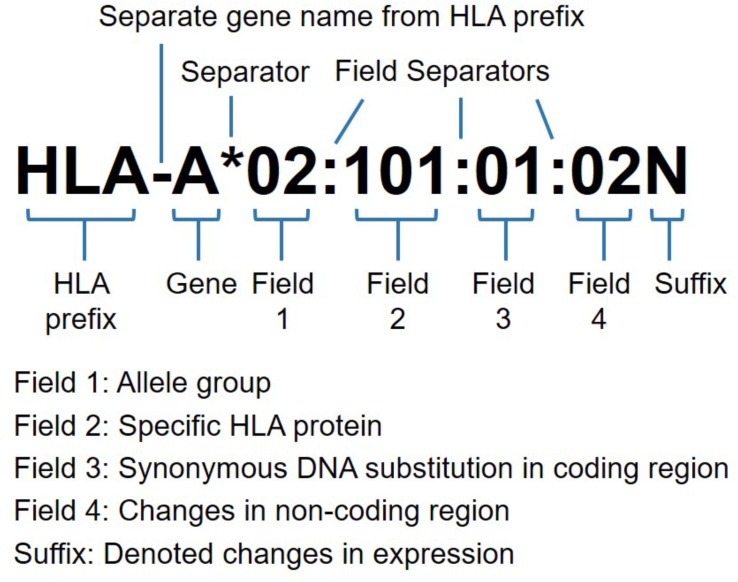
HLA nomenclature with allele resolution at four fields (eight digits). Commonly, the HLA prefix followed by gene name and four fields of the allele. The suffix may be added to an allele to indicate its expression status as low cell surface expression “L”, soluble secreted molecule, “S”, present in the cytoplasm, “C”, aberrant. “A”, questionable, “Q” or not expressed as null alleles “N”. The ambiguous allele typing for identical nucleotide sequences are coded as “G” after first three allele fields, and for identical protein sequences as “P” after two allele fields designation for exons encoding peptide binding domains (exon 2 and 3 for HLA-class I, and exon 2 only for HLA-class II alleles) (adapted from http://hla.alleles.org).

PCR-based HLA typing using sequence-specific primers (SSP), sequence-specific oligonucleotide probes (SSOP), and Sanger sequencing-based typing (SBT) methods have significantly improved HLA typing resolution, however, these methods possess several limitations, including time-consuming protocols, low throughput, unphased data and ambiguity (Wittig et al., 2015). Single nucleotide polymorphism (SNP) based high-throughput DNA microarray studies tend to miss several HLA variants and alternatively use HLA imputation tools (HIBAG, SNP2HLA, HLA^∗^IMP:02, and HLA-check) to infer the classical HLA alleles and amino acid changes using the LD information existing between HLA alleles and SNP markers in the HLA region (Jeanmougin et al., 2017; Karnes et al., 2017). However, higher resolution typing information, including synonymous and non-coding variants of HLA alleles as defined by the third and fourth fields of HLA nomenclature (Figure 1) remains undetected. Such HLA gene variants may be reported in two formats as (a) SNP rsID and (b) with four-field WHO nomenclature (Supplementary Table S2). The nomenclature with both formats (e.g., *HLA-B^∗^08:01* rs4143332) (Fingerlin et al., 2015) may be helpful in designing the routine genotyping assays, such as MassARRAY and Taqman.

## Next-Generation Sequencing Based HLA Typing

HLA polymorphism was first identified and characterized using alloantibodies (antisera) against leukocytes and the microlymphocytotoxicity test was developed for serological typing of HLA antigens (tissue types) in humans (Choo, 2007; Thorsby, 2009). However, since the advent of PCR techniques, DNA-based HLA typing techniques (Shiina et al., 2009; Hosomichi et al., 2015; Robinson et al., 2015; Carapito et al., 2016; Duke et al., 2016; Zhou et al., 2016; Gandhi et al., 2017) have advanced remarkably and rapid progress was driven by the fact that DNA-typing allowed higher HLA resolution and thus provides better diagnostic utility than serotyping. Nowadays, high-resolution HLA typing is the gold standard for HLA-based clinical applications, particularly for hematopoietic stem cell transplant (HSCT) matching and has been also helpful for scientific investigations of the role of HLA in disease pathophysiology.

NGS platforms commonly in use for high-throughput sequencing and massively parallel analysis, including for HLA encompass Ion torrent (Thermo Fisher), Illumina, SOLiD (ABI), PacBio, Oxford Nanopore. In addition to capital costs, they differ in various features, including sequence length and sequencing time; see Supplementary Table S1 for details including the strength and limitations of each platform. The major limitations of NGS owing to cost, sequencing time and technical expertise are also outlined in Supplementary Appendix S1. High-throughput sequencing of the HLA region has been suggested as an integrated tool for accurate detection of SNPs, InDel, CNV, splice site variations, and typing of HLA genes to finer resolution (Shiina et al., 2009; Cao et al., 2013). The technical aspects of NGS based HLA typing have been recently reviewed in detail (Hosomichi et al., 2015; Carapito et al., 2016). Briefly, the steps involved in NGS include: (i) PCR amplification of the target region (complete HLA region, full-length genes, or only certain exons of HLA genes), quantification and enzymatic purification of amplicons; (ii) library preparation by ligation of amplicons to the indexed adaptors, bead based amplicon purification and size selection; (iii) sequencing using NGS platform; and (iv) data analysis using suitable software.

For NGS, the two main clonal techniques specifically targeting the HLA region include, (i) long/mid-range PCR based isolation, and (ii) hybridization-based capture methods; however, achievement of good allelic balance and homogenous coverage along all the target genes remains a major challenge (Hosomichi et al., 2015; Carapito et al., 2016). The combined approach of NGS based advanced methods for increased sequence lengths and advanced bioinformatics tools (Table 1) has potentially emerged as the gold standard for accurate typing of HLA variants (up to fourth field allele resolution) and generation of full-length HLA haplotypes without phase ambiguity. Sequencing of the complete HLA region using HLA sequence libraries is mostly needed to generate a population/ancestry based database and will be useful for the studies of HLA associated diseases (Zhou et al., 2016), whereas HLA typing of the full-length or selected exons of HLA genes is more appropriate for clinical purposes. Furthermore, information from NGS based HLA typing is expected to provide better insight into expression and regulation of HLA genes including epigenetic mechanisms and for an improved understanding of the “HLA-omics” of complex immune diseases (Hosomichi et al., 2015; Carapito et al., 2016).

**Table 1:** HLA typing analysis tools recently reported using next-generation sequencing data (^∗^commercial).

Tools	Information (repository URL)	Reference
HISAT genotype	Assembles full-length sequences of HLA genes including exons and introns and performs HLA typing with alignment from IMGT/HLA database. More sensitive than other short-read aligners (http://ccb.jhu.edu/hisat-genotype)	Kim et al., 2018
xHLA	Fast and accurate HLA typing from short-read NGS data. Useful for protein level typing among both class I and II HLA genes (https://github.com/humanlongevity/HLA)	Xie et al., 2017
HLA-HD	Scans for variation through the entire sequence of HLA gene and accurately determines HLA alleles from NGS data with 6-digit precision. Compatible with low-quality NGS data. (http://www.genome.med.kyoto-u.ac.jp/HLA-HD)	Kawaguchi et al., 2017
HLAscan	An alignment-based program that determines haplotypes taking read distribution into account. Helpful for variant calling in highly polymorphic regions (http://www.genomekorea.com/display/tools/HLAscan)	(Ka et al., 2017)
^∗^HLA twin (Omixon)	Combines two independent computational algorithms to ensure high confidence in allele calling. Consensus sequence and typing results reported as Histoimmunogenetics Markup Language (HML) format	–
^∗^NGSengine (GenDx)	Single button analysis for NGS data provides genotyping results with minimal editing	–

With advancement of high-throughput HLA typing based on NGS, there has been an enormous expansion of the numbers of new HLA sequences and new alleles submitted to HLA databases (13,324 class I, 4857 class II and 182 other non-HLA alleles in the evolving Immuno Polymorphism Database-ImMunoGeneTics/HLA ‘IPD-IMGT/HLA ^1^′ (Robinson et al., 2015; Marsh and WHO Nomenclature Committee for Factors of the HLA System, 2018). The common and well-documented ‘CWD’ catalog for HLA-class I and II alleles has been maintained to update the HLA alleles found at known frequencies (common ‘C’) or replicated using sequence-based typing and HLA haplotype data (well-documented ‘WD’)^2^ (CWD v2.0.0) (Mack et al., 2013). The CWD categories also extend to ‘G’ and ‘P’ designations for different alleles with identical nucleotide and protein sequences, respectively (Figure 1). However, even with NGS HLA typing, challenges still remain with respect to phasing of polymorphisms leading to ambiguous results. The challenges could be compounded by PCR dropout or allelic imbalance, libraries with fragments sizes too small to achieve phasing and an inability to sequence selected HLA regions, such as exon 1 of the *DPB1* and *DRB1* genes (Carapito et al., 2016; Duke et al., 2016; Gandhi et al., 2017).

## HLA and Disease Associations

The HLA region is associated with more than 100 multifactorial, complex diseases mainly of inflammatory and autoimmune pathogenesis. These diseases include coeliac disease, psoriasis, rheumatoid arthritis, type-1 diabetes, systemic lupus erythematosus, lung diseases (e.g., asthma and sarcoidosis), infectious diseases (e.g., HIV, hepatitis, leprosy, tuberculosis) and other conditions such as malignancies and neuropathies (Trowsdale and Knight, 2013; Carapito et al., 2016). In most cases it has been challenging to identify the principal genetic association, primarily for the following reasons: high-density of HLA genes, strong LD and effects of multiple HLA loci. However, use of NGS to fine map the HLA genes for unreported alleles and unambiguous haplotypes has already been helpful in resolving the complexity of the HLA-associated diseases and their clinical subtypes. For example, in psoriasis, new associations of *HLA-DPB1* and *BTNL2* genes and five loci among *HLA-C*, *-B*, *-DPB1*, and *BTNL2* were reported using NGS (Zhou et al., 2016). Distinct HLA associations were reported for the overall risk of psoriasis vulgaris and risk of its specific subphenotypes, such as psoriatic arthritis and cutaneous psoriasis (Okada et al., 2014).

In future, allelic resolution of HLA genes obtained by NGS will be helpful in studying the effect of variant interactions in disease risk, in selecting the participants for prevention or intervention trials (Zhou et al., 2016) and in deciphering antigen presentation mechanisms and T cell responses in chronic autoimmune disorders, such as rheumatoid arthritis (Sakurai et al., 2018). There are further important applications of NGS based HLA typing in biomedicine, namely (i) HLA-matching of donor-recipient pairs for HSCT, including HLA typing for registries of unrelated bone-marrow donors, and (ii) characterisation of individual response to drug therapy including pharmacogenetics. Further (iii) NGS will facilitate the novel strategies based on epitope matching in solid organ transplantation.

## The Role of HLA in Sarcoidosis

Sarcoidosis is a multi-systemic disease mainly affecting the lungs. It exhibits complex behavior, including the presence of multiple phenotypes, variable disease onset, and clinical course of the disease (Valeyre et al., 2014; Patterson and Chen, 2017). Sarcoidosis is believed to be the outcome of inefficient processing of yet unknown antigenic peptide(s) and their presentation by HLA with a subsequent aberrant immune response in the affected organs. This process – represented by the molecular triad (antigen presenting cells with HLA/antigen peptide/TCRs on T cells) – is further affected by the combined interaction of the genetic background of an individual and environmental pathogenic factors (Kishore and Petrek, 2013; Moller et al., 2017). Initial investigations of the role of HLA in sarcoidosis were performed by serological typing as exemplified by a report of HLA-B8 and -B13 associations with sarcoidosis in the Czech population (Levin et al., 2013). The concept of larger association studies investigating common risk HLA alleles for more than one population (ethnicity) has been demonstrated by a joint study with Czech and Italian patient cohorts, which also analyzed diverse sarcoidosis clinical phenotypes (Martinetti et al., 1995). Later, with the advancement of DNA-based HLA-typing, the extended alleles were determined as HLA-B^∗^08:01, -B^∗^08:04, -B^∗^13:01, and -B^∗^13:02 (Robinson et al., 2015; Marsh and WHO Nomenclature Committee for Factors of the HLA System, 2018); and other HLA variants implicated in sarcoidosis were identified in both European and U.S. studies (Casanova et al., 2015; Grunewald et al., 2016). The immunogenic determinants at 6p21.3, including the HLA region, reported in sarcoidosis using genome-wide association and candidate gene studies (Kishore and Petrek, 2013; Fingerlin et al., 2015; Moller et al., 2017) are briefly summarized in the following paragraph.

In the HLA region, candidate loci for sarcoidosis include HLA genes *HLA-A, -B, -DPB1, -DQB1, -DRB1, -DRB3*; and non-HLA genes *BTNL2*, *C4*, *C6orf10*, *HSPA1L*, *LTA, NOTCH4*, *TAP2*, *TNF*, and *VEGF*. The linkage of class III genes, such as *TNF* and *LTA* has been demonstrated with class I *HLA-B* and class II *HLA-DRB1*). These gene variants have been shown to associate with sarcoidosis phenotypes and sarcoidosis disease course (Grunewald et al., 2015). Association of variants *C6orf10*^∗^rs3129927 and *HLA-DRA*^∗^rs3135394 was reported for Löfgren’s syndrome (LS) – a particular phenotype of more benign disease. By contrast, *BTNL2*^∗^rs2076530 and *HLA-DRA*^∗^rs3129882 was associated with the non-LS sarcoidosis (Rivera et al., 2016; Wolin et al., 2017) (Supplementary Table S2). To mention the further associations, *HLA-DRB1^∗^01/^∗^04* allelic groups have been associated with decreased sarcoidosis risk, *HLA-B^∗^08* with increased risk, *HLA-DRB1^∗^03:01* with LS and acute disease onset, *HLA-DRB1^∗^12/^∗^14* with non-LS, *HLA-DRB1^∗^14:01* with chronic disease, HLA*-DRB1^∗^04/^∗^15* with the extrapulmonary involvement, *HLA-DQB1^∗^0602* with disease progression, haplotype *HLA-DRB1^∗^04:01*-*DPB1^∗^04:01* with disease resolution (Foley et al., 2001; Kishore and Petrek, 2013; Fingerlin et al., 2015; Moller et al., 2017). For details please refer to Supplementary Table S2. In addition to HLA, other genes with apoptotic, enzymatic, regulatory, immune and inflammatory functions have been implicated as candidate loci in sarcoidosis (Sato et al., 2010). These include e.g., *ANXA11, ACE, CCR2, CCR5, IL1A, IL23R, NOD2*, and *NRAMP1* (McGrath et al., 2001; Hutyrová et al., 2002; Petrek et al., 2002; Kishore and Petrek, 2013) (Supplementary Table S3), however, detailed discussion of these associations is beyond the scope of this mini-review.

To date, most information available concerning the involvement of the HLA sequence variation in sarcoidosis has been derived from low resolution HLA typing, to the first or maximally second field resolution. Higher resolution typing, which is now possible, particularly for non-coding sequences, will be helpful to pinpoint the specific contribution of particular HLA variants to disease susceptibility and development. In this regard, NGS based HLA typing promises to provide a valuable resource for the precise designation of HLA alleles and/or haplotypes. In near future it may be utilized for further dissecting the role of HLA variation in disease susceptibility and the development of distinct phenotypes, ranging from benign Löfgren’s syndrome to progressing sarcoidosis. Thus, precise HLA typing by NGS should not only validate the previous findings and extend our knowledge of the genetic basis of sarcoidosis, but also promises to provide an insight into mechanisms of disease progression. Translational applications such as usage of HLA data obtained by NGS in clinical patient management, e.g., for prognosis, may be envisaged.

## NGS Based HLA Typing in Population Studies and Its Application for Investigating HLA Disease Association

NGS based HLA typing has advantages for population studies, which are important not only for population genetics itself but also for the interpretation of disease association studies or utilization of transplantation donor registries. In all these applications, NGS can fill gaps in the existing data or could establish new population-specific HLA databases with improved accuracy (Yin et al., 2016). For example, population studies giving the prevalence of HLA alleles (CWD catalog) provide a reference to aid resolution of typing ambiguities in tissue transplantation or to determine the presence and prevalence of any HLA allele in different human populations (Mack et al., 2013; Sanchez-Mazas et al., 2017). This information can be updated and improved by NGS HLA typing. One of the first applications of NGS in population studies has been a report of a database of the complete HLA region in a Han Chinese population (Zhou et al., 2016). In a Saudi cohort of healthy individuals, the allele and haplotype frequencies of HLA genes *(HLA-A, -B, -C, -DRB1*, and *-DQB1*) were reported with improved resolution using NGS HLA typing (Hajeer et al., 2013). At targeted HLA gene level, NGS based *HLA-E* gene diversity was evaluated in West-Africans (Castelli et al., 2015). The current level of polymorphism in the HLA region characterized for both European and American populations is mostly at the second field level (Mack et al., 2013; Sanchez-Mazas et al., 2017). This is expected to be characterized with greater detail using NGS, as has recently been reported for British and Argentinian populations (Davey et al., 2017; Hurley et al., 2018).

In the context of disease association studies, it has been suggested that risk conferred by HLA can differ between distinct populations (Ortiz-Fernández et al., 2014; Martínez-Ojinaga et al., 2018). A population-wise descriptive list of *HLA* alleles (at low resolution) as increased or decreased risk variants in disease phenotypes and disease progression in sarcoidosis has been outlined (Kishore and Petrek, 2013; Fingerlin et al., 2015). Coeliac disease represents another example of a HLA associated disease with various European populations. This disease associates with *HLA-DQA* and -*DQB* genes and about 96% of European coeliac patients carry the *HLA-DQ2.5* heterodimer (*DQA1^∗^05*-*DQB1^∗^0201*) (Karell et al., 2003; Martínez-Ojinaga et al., 2018; Núñez et al., 2018) and in some reports differed among non-Europeans, for e.g., in Brazilians and Iranians, -DQ2.2 was not associated with coeliac disease (Rostami-Nejad et al., 2014; Selleski et al., 2018). Also, the differential HLA basis of the *GIMAP* locus, which encoded proteins involved in the development of T and B lymphocytes, and Behçet’s disease was described in Asian and European populations (Ortiz-Fernández et al., 2014).

An improved knowledge of HLA population genetics is therefore of great importance for investigations of particular HLA allele and/or haplotype disease associations across different ethnic groups. For this purpose, the HLA databases such as allelefrequency.net (González-Galarza et al., 2015) or specific allele and/or haplotype catalogs providing data on common alleles and/or haplotypes and genetic architecture of HLA in the population should be exploited. Their update with allele resolution HLA typing by NGS will provide valuable information including previously unmapped regions (introns, expanded exons, and other gene regulatory sequences) and will be helpful for further understanding the role of the implicated HLA variants in disease susceptibility and modification of clinical outcome. In this context, general guidelines such as STrengthening the REporting of Genetic Association studies (STREGA) and the STrengthening the REporting of Immunogenomic Studies (STREIS) statement could also be applied for analyses of HLA associations (Hollenbach et al., 2011). Further efforts in this area are underway, greatly stimulated by the activities of the 17th International HLA and Immunogenetics Workshop ‘IHIW,’ which evolved around NGS HLA typing and its applications, with a centralized approach for data analysis and management, including data collection, sharing and storage (Chang et al., 2017). The details for ongoing projects and updates related to HLA population genetics, such as EFI-PG, EUROSTAM, HLA-NET, and AHPD can be accessed at https://hla-net.eu/projects/.

## Regulation of HLA Genes by Epigenetic Mechanisms

The rapidly evolving field of epigenetics deciphers the complex crosstalk between genetic factors and environmental exposure. The main epigenetic mechanisms include DNA methylation, histone modifications and non-coding RNA (microRNA, miRNA). In the context of HLA, non-coding sequences from HLA regions, particularly the regulatory enhancers, promoter and untranslated regions (5’- and 3’-UTRs) could form a repertoire for non-genetic factors acting as epigenetic regulators of HLA genes. Primarily, the regulatory elements have differential CpG methylation and miRNA binding sites, while histone modifications are derived by specific enzymes or protein complexes. Interestingly, HLA was among the first regions explored for methylation profiling, the HLA region can be analyzed for both genetic and epigenetic features (Tomazou et al., 2008). The evidence for involvement of DNA methylation in HLA gene regulation is exemplified by the observation that high methylation of the *HLA-A* promoter region correlated with a reduced level of gene expression (Ramsuran et al., 2015). Methylation also exerts long gene distance impacts, as three CpG sites in the *HLA-DQB1* coding region are associated (non-linkage) with the promoter region of *HLA-F* (Esterhuyse et al., 2015). Furthermore, the hypermethylated *HLA-DQB1* gene in tuberculosis patients was associated with reduced expression of HLA-class II genes (Esterhuyse et al., 2015). Histone acetylation and methylation can activate *HLA-DRA* gene expression through multiple complexes of histone lysine acetyltransferase (KAT) and histone lysine methyltransferase (KMT) respectively, in a class II transactivator (CIITA) independent or dependent manner (Choi and Boss, 2012). The UTRs sequence variation may be a predictor of the genetic predisposition of an individual to express different levels of *HLA-G* as reported in several studies. In this context, *HLA-G 5*’*-UTR* methylation was associated with *HLA-G* gene silencing (Carosella et al., 2015) and *HLA-G 3*’*-UTR* with a common SNP rs1063320 was reported to modulate binding of miRNA-148/152 family and suppress soluble *HLA-G* expression in asthma (Naidoo et al., 2015).

Finally, in the context of HLA there have been reports of the involvement of yet another epigenetic mechanism – short-sequence non-coding miRNAs. Firstly, miRNA expression itself may be influenced by the presence of SNPs (miR-SNP) in the seed domain, which eventually modulates the target gene expression (Kishore et al., 2014). Similarly, genetic variation in the distal *HLA-A 3*’*-UTR* (100 bp insertion) affecting RNA binding protein Syncrip was shown to modulate the post-transcriptional expression of the HLA-A protein (Kulkarni et al., 2017). A concerted effect of methylation and genomic variation (14 bp insertion) in *HLA-G 3*’*-UTR* was also demonstrated for *HLA-G* regulation (Verloes et al., 2017). Secondly, an expanded role for HLA genes in regulating other immune responses via miRNA interaction was demonstrated by inhibition of *HLA-B* intron-encoded miR-6891-5p that impacts the expression of several transcripts, including I gA, affecting a large number of metabolic and immune response pathways (Chitnis et al., 2017). It is conceivable that investigations of miRNA in the context of HLA will continue, as exemplified e.g., by sequence characterization of novel and haplotype-specific miRNA transcripts for complete *HLA* region using deep RNA sequencing (Clark et al., 2018).

## Conclusion

HLA typing has already benefited and will continue to benefit from NGS applications. Comprehensive and unambiguous characterisation of extensive polymorphisms in the HLA region by NGS will contribute to progress in the area of HLA and disease associations by precise identification of these associations at the allelic level and by utilizing exact data from NGS-based population genetic studies. In sarcoidosis, the translation of expected developments to clinical practice will aim at defining new genetic markers for particular disease phenotypes and at using knowledge on the nature of yet unknown peptides presented by HLA molecules for understanding disease mechanisms, resulting in development of specifically tailored therapies. In our opinion, dynamic developments in NGS methodologies and linked bioinformatic analyses will ultimately expand clinical applications of HLA typing beyond traditional areas of transplantation and immunogenetics. Understanding the molecular mechanisms of sarcoidosis using these novel approaches represents just a small piece at the start of these endeavors.

## Author Contributions

MP and AK contributed to the conception and writing of this review. Both authors have reviewed and approved the submitted work.

## Conflict of Interest Statement

The authors declare that the research was conducted in the absence of any commercial or financial relationships that could be construed as a potential conflict of interest.

## Footnotes

1 www.ebi.ac.uk/ipd/imgt/hla/stats.html; http://hla.alleles.org/inc/images/graph_hires.png

2 http://igdawg.org/cwd.html
