# Supplementary material for: Next-Generation Sequencing Based HLA Typing: Deciphering Immunogenetic Aspects of Sarcoidosis
Source: Front Genet. 2018 Oct 25;9:503. doi: 10.3389/fgene.2018.00503 (PMC6210504; doi:10.3389/fgene.2018.00503)
Supplement: Supplementary file 1 [file Data_Sheet_1.docx]

*Supplementary Material*

Next-generation sequencing based HLA typing: deciphering immunogenetic aspects of sarcoidosis

Amit Kishore^1^, Martin Petrek^1*^

^1^Department of Pathological Physiology, Faculty of Medicine and Dentistry, Palacky University, Olomouc 77515, Czech Republic

*** Correspondence:**Martin Petrek
[martin.petrek@fnol.cz](mailto:martin.petrek@fnol.cz)

**Appendix S1:**

**Limitations of Next-generation Sequencing**

Although unambiguous typing is ‘the gold standard’ for HLA typing, there are several drawbacks to this approach: (i) Cost of the instrument (for example, US$ 80000 for Ion Torrent PGM, US$ 690000 for HiSeq 2000, and US$ 695000 for PacBio); and the cost to type a sample (US$ 350 for Ion Torrent PGM, US$ 6000 for HiSeq 2000, and US$ 300 for PacBio) and/or HLA specific typing kits and software. (ii) Time consuming, particularly for platforms with higher accuracy results; for examples, it may take up to 10 days to sequence the samples using HiSeq 2000. (iii) Technical expertise is required for the sample and library preparations as well as clonal PCR (1,2). However, with advancement in the technology, efforts are being made for NGS being more affordable with the decrease in cost and simplified methods.

**Supplemental tables**

**Table S1: Sequencing platforms, their principles and (dis)advantages.**

| **Company** | **Instrument** | **Sequencing principle** | **Throughput range (Gb)** | **Read length (bp)** | **Strength** | **Weakness** |
| --- | --- | --- | --- | --- | --- | --- |
| Applied Biosystems (ABI) | ABI 3500/3730 | Sanger method | 0.0003 | Up to 1000 | Read accuracy and length | Cost and throughput |
| Roche/454 | FLX | Pyrosequencing | ~20 Mb | 110 | (Discontinued from 2016) | |
| ABI (Life Technologies; now Thermo Fisher Scientific) | SOLiD | Ligation sequencing | 3-30 | 35-85 | Accuracy | Run time |
| Illumina | MiniSeq | Sequencing by synthesis | 1.7–7.5 | 1x75 to x150 | Low initial investment | Run and read length |
|  | MiSeq |  | 0.3-15 | 1x36 to 2x300 | Read length, scalability | Run length |
|  | NextSeq |  | 10-120 | 1x75 to 2x50 | Throughput | Run and read length |
|  | HiSeq (2500) |  | 10-1000 | x50 to x250 | Read accuracy, throughput | High initial investment, run |
|  | NovaSeq 5000/6000 |  | 2000-6000 | 2x50 to x150 | Read accuracy, throughput | High initial investment, run |
| Ion Torrent (Thermo Fisher Scientific) | Personal genome machine (PGM) | Polymerase sequencing by synthesis. Detection by semiconductor technology | 0.08-2 | Upto 400 | Read length, speed | Throughput, homopolymers |
|  | S5 |  | 0.6-15 | Upto 400 | Read length, speed | Homopolymers |
|  | Proton |  | 10-15 | Upto 200 | Speed, throughput | Homopolymers |
| Pacific Biosciences (PacBio) | RS Platform | Signal with incorporation of phosphonucleotides into DNA. Single molecule real time detection. | 0.5-1 | Upto 60 kb | Read length, speed (Average 10 kb, N50 20 kb) | High error rate and initial |
|  | Sequel |  | 5-10 | ” | ” | High error rate |
| Oxford Nanopore Technology (ONT) | MinIon | Electronic identification of nucleotides using protein nanopores | 0.1-1 | Upto 100 kb | Read length, portability | High error rate, run length |

The table is modified and adapted from a recent review (3). The table listed represent a selection, some of the less common/available platforms could not be mentioned.

**Table S2:** HLA alleles, variants and haplotypes associated with sarcoidosis and its particular phenotypes.

| **HLA-class** | **Gene** | **alleles / variant** | **Plausible association or functional significance** | **Associated population** | **Reference** |
| --- | --- | --- | --- | --- | --- |
| Class I | HLA-A | *01 | Susceptible for sarcoidosis | Czech | (4–6) |
| Class I | HLA-B | *08:01 | Susceptible | Multiple | (4,5,7) |
| Class II | HLA-DPB1 | *04:02 | Lower risk or protective | Finnish | (4,8) |
|  |  | rs9277357 | Non-Löfgren’s syndrome ‘non-LS’ | European cohorts | (9) |
| Class II | HLA-DQB1 | *02:01 | Löfgren’s syndrome ‘LS’, protective | Multiple | (4,5) |
|  |  | *05:01 | Lower risk | Multiple | (4,10) |
|  |  | *05:03 | Increased risk of non-LS | Dutch | (7) |
|  |  | *06:02 | Susceptible/disease progression | Multiple | (4,5) |
| Class II | HLA-DQA1 | rs2187668 | LS | European cohorts | (9) |
| Class II | HLA-DRA | rs3135394 | LS | European cohorts | (9) |
|  |  | rs3129882 | Non-LS | European cohorts | (9) |
|  | HLA-DRA 3’UTR | rs3177928 | Non-LS | European cohorts | (11) |
|  | (HLA-DRA, HLA-DRB5) | rs1964995 | Non-LS | European cohorts | (9) |
| Class II | HLA-DRB1 | *01, *04 | Lower risk | Multiple | (5,7,10) |
|  |  | *03:01 | LS; acute onset; good prognosis | Multiple | (5,7,8,10) |
|  |  | *11:01 | Susceptible | AA and EA | (5) |
|  |  | *12:01, *14:01 | Chronic sarcoidosis; increased risk of Non-LS | AA and EA | (4,7) |
|  |  | *15:01 | LS | Polish | (4,7) |
|  |  |  | Risk of Non-LS | AA and EA | (4,7) |
|  |  | rs3830135 | Non-LS | European cohorts | (9) |
| Class II | HLA-DRB3 | *01:01 | Non-LS and disease progression | AA and EA | (4,5,7) |
| HLA | HLA-DRB1*04:01-DPB1*04:01 | | Resolved sarcoidosis | Finnish | (4) |
| haplotype | HLA-DRB1*15:01-DQB1*06:02 | | Strong marker for severe pulmonary sarcoidosis | Dutch | (4) |
|  | HLA-DRB1*04-DQB1*03:01 | | Risk for disease manifestation; overall lower risk | British, Japanese | (4,10) |
|  | HLA-DRB1*03:01-DRB3*01:01 | | Pulmonary sarcoidosis | Multiple | (4) |
|  | HLA-DRB1*03:01-DQB1*02:01 | | LS | Dutch | (4,7,10) |
|  | HLA-DQB1*06:02-DRB1*15 | | Increased risk of Non-LS | Dutch | (7) |

Abbreviations as AA, African Americans; EA, Americans of European ancestry; HLA, human leukocyte antigen (major histocompatibility complex); LS, Löfgren’s syndrome; rs, reference SNP; UTR, untranslated region.

**Table S3:** Non-HLA gene variants associated with sarcoidosis and its subtypes with emphasis on Löfgren’s syndrome (selection).

| **Gene** | **variant** | **Putative functional significance** | **Associated population** | **Reference** |
| --- | --- | --- | --- | --- |
| BTNL2^#^ | rs3117099 | Löfgren’s syndrome ‘LS’ | British and Dutch | (7,12) |
|  | rs2076530 | Increased risk of Non-LS | British, Dutch, and Portuguese | (5,7,12,13) |
|  | rs2294878 | Increased risk of Non-LS | British and Dutch | (7,12) |
| TNF^#^ | rs1800629 | LS | Czech, Dutch, EA, German and Polish | (4,7,14) |
| LTA^#^ | rs909253 | LS | Polish, EA females | (7) |
|  | rs2239704 | Non-LS | European cohorts | (9) |
| C6orf10^#^ | rs3129927 | LS | European cohorts | (9) |
| (C6orf10, BTNL2)^#^ | rs3129950 | LS | European cohorts | (9) |
| BTNL2^#^ | rs2076530 | Non-LS | European cohorts | (9) |
| (BTNL2, HLA-DRA)^#^ | rs3129843 | LS | European cohorts | (11) |
| (ATF6B, CREBL1)^#^ | rs3130288 | LS | European cohorts | (9) |
| TAP2^#^ | rs3819717 | Non-LS | European cohorts | (9) |
| ANXA11 | rs1049550 | Lower risk; Non-LS | Czech | (15) |
|  | rs61860052, rs4377299 | Susceptibility | AA and EA | (16) |
| ACE | rs121912703 | Elevated serum ACE in sarcoidosis | Dutch | (17) |
|  | I/D variant | Increased risk for ID and DD genotypes | Czech | (18) |
| BTNL2 | rs2076530 | Susceptibility | German | (19) |
| CCR2 | Val64Ile | LS and lower risk | Czech | (20) |
| CCR5 | Delta 32 allele | Disease persistent | Czech | (21,22) |
| IL1A | CC genotype; C allele at - 889 | Susceptibility | Czech | (23) |
| IL23R | rs11209026 | Acute and chronic sarcoidosis | German | (24) |
| NOD2 | Arg334Trp | Early-onset sarcoidosis | Japanese | (25) |
| SLC11A1 | 5’-(GT)n allele 2/3; INT4 G/C | Susceptibility | Turkish | (26) |

^#^ Non-HLA variants in the HLA region; intergenic variants are mentioned in parenthesis; abbreviations as AA, African Americans; ACE, angiotensin I converting enzyme; ANXA11, annexin A11; ATF6B, activating transcription factor 6 beta; BTNL2, butyrophilin-like 2 (HLA-class II associated); C6orf10, chromosome 6 open reading frame 10 ; CCR2, C-C chemokine receptor 2; CCR5, C-C chemokine receptor 5; CREBL1, CAMP responsive element binding protein-like 1; EA, Americans of European ancestry; HLA, human leukocyte antigen (major histocompatibility complex); IL1A, interleukin 1 alpha; IL23R, interleukin 23 receptor; LTA, lymphotoxin alpha; NOD2, nucleotide binding oligomerization domain containing 2; SLC11A1, solute carrier family 11 member 1; rs, reference sequence; TAP2, transporter 2, ATP binding cassette subfamily B member; TNF, tumor necrosis factor.

**References**

1. Quail MA, Smith M, Coupland P, Otto TD, Harris SR, Connor TR, Bertoni A, Swerdlow HP, Gu Y. A tale of three next generation sequencing platforms: comparison of Ion Torrent, Pacific Biosciences and Illumina MiSeq sequencers. *BMC Genomics* (2012) **13**: doi:10.1186/1471-2164-13-341

2. Shendure J, Ji H. Next-generation DNA sequencing. *Nat Biotechnol* (2008) **26**:1135–1145. doi:10.1038/nbt1486

3. Besser J, Carleton HA, Gerner-Smidt P, Lindsey RL, Trees E. Next-generation sequencing technologies and their application to the study and control of bacterial infections. *Clin Microbiol Infect* (2018) **24**:335–341. doi:10.1016/j.cmi.2017.10.013

4. Kishore A, Petrek M. Immunogenetics of Saarcoidosis. *Int Trends Immun* (2013) **1**:43–53.

5. Sah BP, Iannuzzi MC. “Genetic Factors Involved in Sarcoidosis,” in *Sarcoidosis* (InTech), 53–77. doi:10.5772/55116

6. Martinetti M, Tinelli C, Kolek V, Cuccia M, Salvaneschi L, Pasturenzi L, Semenzato G, Cipriani A, Bartova A, Luisetti M. “The sarcoidosis map”: a joint survey of clinical and immunogenetic findings in two European countries. *Am J Respir Crit Care Med* (1995) **152**:557–64. doi:10.1164/ajrccm.152.2.7633707

7. Fingerlin TE, Hamzeh N, Maier LA. Genetics of Sarcoidosis. *Clin Chest Med* (2015) **36**:569–84. doi:10.1016/j.ccm.2015.08.002

8. Wennerström A, Pietinalho A, Lasota J, Salli K, Surakka I, Seppänen M, Selroos O, Lokki M-L. Major histocompatibility complex class II and BTNL2 associations in sarcoidosis. *Eur Respir J* (2013) **42**:550–3. doi:10.1183/09031936.00035213

9. Rivera N V, Ronninger M, Shchetynsky K, Franke A, Nöthen MM, Müller-Quernheim J, Schreiber S, Adrianto I, Karakaya B, van Moorsel CHM, et al. High-Density Genetic Mapping Identifies New Susceptibility Variants in Sarcoidosis Phenotypes and Shows Genomic-driven Phenotypic Differences. *Am J Respir Crit Care Med* (2016) **193**:1008–1022.

10. Sato H, Woodhead FA, Ahmad T, Grutters JC, Spagnolo P, van den Bosch JMM, Maier LA, Newman LS, Nagai S, Izumi T, et al. Sarcoidosis HLA class II genotyping distinguishes differences of clinical phenotype across ethnic groups. *Hum Mol Genet* (2010) **19**:4100–4111. doi:10.1093/hmg/ddq325

11. Wolin A, Lahtela EL, Anttila V, Petrek M, Grunewald J, van Moorsel CHM, Eklund A, Grutters JC, Kolek V, Mrazek F, et al. SNP Variants in Major Histocompatibility Complex Are Associated with Sarcoidosis Susceptibility-A Joint Analysis in Four European Populations. *Front Immunol* (2017) **8**:422. doi:10.3389/fimmu.2017.00422

12. Spagnolo P, Sato H, Grutters JC, Renzoni EA, Marshall SE, Ruven HJT, Wells AU, Tzouvelekis A, Van Moorsel CHM, Van Den Bosch JMM, et al. Analysis of BTNL2 genetic polymorphisms in British and Dutch patients with sarcoidosis. *Tissue Antigens* (2007) **70**:219–227. doi:10.1111/j.1399-0039.2007.00879.x

13. Morais A, Lima B, Peixoto MJ, Alves H, Marques A, Delgado L. BTNL2 gene polymorphism associations with susceptibility and phenotype expression in sarcoidosis. *Respir Med* (2012) **106**:1771–1777. doi:10.1016/j.rmed.2012.08.009

14. Mrazek F, Holla LI, Hutyrova B, Znojil V, Vasku A, Kolek V, Welsh KI, Vacha J, du Bois RM, Petrek M. Association of tumour necrosis factor-alpha, lymphotoxin-alpha and HLA-DRB1 gene polymorphisms with Löfgren’s syndrome in Czech patients with sarcoidosis. *Tissue Antigens* (2005) **65**:163–71. doi:10.1111/j.1399-0039.2005.00370.x

15. Mrazek F, Stahelova A, Kriegova E, Fillerova R, Zurkova M, Kolek V, Petrek M. Functional variant ANXA11 R230C: True marker of protection and candidate disease modifier in sarcoidosis. *Genes Immun* (2011) **12**:490–494. doi:10.1038/gene.2011.27

16. Levin AM, Iannuzzi MC, Montgomery CG, Trudeau S, Datta I, McKeigue P, Fischer A, Nebel A, Rybicki BA. Association of ANXA11 genetic variation with sarcoidosis in African Americans and European Americans. *Genes Immun* (2013) **14**:13–8. doi:10.1038/gene.2012.48

17. Stouten K, van de Werken M, Tchetverikov I, Saboerali M, Vermeer HJ, Castel R, Verheijen FM. Extreme elevation of serum angiotensin-converting enzyme (ACE) activity: always consider familial ACE hyperactivity. *Ann Clin Biochem* (2014) **51**:289–93. doi:10.1177/0004563213489812

18. McGrath DS, Foley PJ, Petrek M, Izakovicova-Holla L, Kolek V, Veeraraghavan S, Lympany PA, Pantelidis P, Vasku A, Wells AU, et al. ACE Gene I/D Polymorphism and Sarcoidosis Pulmonary Disease Severity. *Am J Respir Crit Care Med* (2001) **164**:197–201. doi:10.1164/ajrccm.164.2.2011009

19. Pacheco Y, Calender A, Israël-Biet D, Roy P, Lebecque S, Cottin V, Bouvry D, Nunes H, Sève P, Pérard L, et al. Familial vs. sporadic sarcoidosis: BTNL2 polymorphisms, clinical presentations, and outcomes in a French cohort. *Orphanet J Rare Dis* (2016) **11**:165. doi:10.1186/s13023-016-0546-4

20. Petrek M, Drabek J, Kolek V, Zlamal J, Welsh KI, Bunce M, Weigl E, Du BR. CC chemokine receptor gene polymorphisms in Czech patients with pulmonary sarcoidosis. *Am J Respir Crit Care Med* (2000) **162**:1000–1003.

21. Spagnolo P, Renzoni EA, Wells AU, Copley SJ, Desai SR, Sato H, Grutters JC, Abdallah A, Taegtmeyer A, du Bois RM, et al. C-C chemokine receptor 5 gene variants in relation to lung disease in sarcoidosis. *Am J Respir Crit Care Med* (2005) **172**:721–8. doi:10.1164/rccm.200412-1707OC

22. Petrek M, Gibejova A, Drabek J, Mrazek F, Kolek V, Weigl E, du Bois RM. CC chemokine receptor 5 (CCR5) mRNA expression in pulmonary sarcoidosis. *Immunol Lett* (2002) **80**:189–93. Available at: http://www.ncbi.nlm.nih.gov/pubmed/11803051 [Accessed May 7, 2018]

23. Hutyrová B, Pantelidis P, Drábek J, Zůrková M, Kolek V, Lenhart K, Welsh KI, Du Bois RM, Petrek M. Interleukin-1 Gene Cluster Polymorphisms in Sarcoidosis and Idiopathic Pulmonary Fibrosis. *Am J Respir Crit Care Med* (2002) **165**:148–151. doi:10.1164/ajrccm.165.2.2106004

24. Fischer A, Nothnagel M, Franke A, Jacobs G, Saadati HR, Gaede KI, Rosenstiel P, Schürmann M, Müller-Quernheim J, Schreiber S, et al. Association of inflammatory bowel disease risk loci with sarcoidosis, and its acute and chronic subphenotypes. *Eur Respir J* (2011) **37**:610–6. doi:10.1183/09031936.00049410

25. Takeuchi Y, Shigemura T, Kobayashi N, Kaneko N, Iwasaki T, Minami K, Kobayashi K, Masumoto J, Agematsu K. Early diagnosis of early-onset sarcoidosis: a case report with functional analysis and review of the literature. *Clin Rheumatol* (2017) **36**:1189–1196. doi:10.1007/s10067-017-3544-6

26. Akçakaya P, Azeroglu B, Even I, Ates O, Turker H, Ongen G, Topal-Sarikaya A. The functional SLC11A1 gene polymorphisms are associated with sarcoidosis in Turkish population. *Mol Biol Rep* (2012) **39**:5009–16. doi:10.1007/s11033-011-1297-x
